# When Healthcare Professionals Use AI: Exploring Work Well-Being Through Psychological Needs Satisfaction and Job Complexity

**DOI:** 10.3390/bs15010088

**Published:** 2025-01-18

**Authors:** Weiwei Huo, Qiuchi Li, Bingqian Liang, Yixin Wang, Xuanlei Li

**Affiliations:** 1 SILC Business School, Shanghai University, Shanghai 200444, China; huoweiwei@shu.edu.cn (W.H.); 15026865177@163.com (Q.L.); wyx2091@shu.edu.cn (Y.W.); 2 School of Management, Fudan University, Shanghai 200433, China; 15298880351@163.com

**Keywords:** use of AI, psychological needs satisfaction, work well-being, job complexity, self-determination theory

## Abstract

This study examines how the use of artificial intelligence (AI) by healthcare professionals affects their work well-being through the satisfaction of basic psychological needs, framed within Self-Determination Theory. Data from 280 healthcare professionals across various departments in Chinese hospitals were collected, and the hierarchical regression and regression were analyzed to assess the relationship between the use of AI, psychological needs satisfaction (autonomy, competence, and relatedness), and their work well-being. The results reveal that the use of AI enhances work well-being indirectly by increasing the satisfaction of these psychological needs. Additionally, job complexity serves as a boundary condition that moderates the relationship between the use of AI and work well-being. Specifically, job complexity weakens the relationship between the use of AI and the satisfaction of autonomy and competence, while having no significant effect on the relationship between the use of AI and the satisfaction of relatedness. These findings suggest that the impact of the use of AI on healthcare professionals’ well-being is contingent on job complexity. This study highlights that promoting healthcare professionals’ well-being at work in the context of AI adoption requires not only technological implementation but also ongoing adaptation to meet their evolving psychological needs. These insights provide a theoretical foundation and practical guidance for integrating AI into healthcare to support the well-being of healthcare professionals.

## 1. Introduction

In recent years, global healthcare systems have confronted unprecedented challenges, with healthcare professionals experiencing intensifying work stress amid persistent workforce shortages (Bamforth et al., 2023; Ahmed, 2019). Meanwhile, medical artificial intelligence (AI) has demonstrated substantial advancements across multiple domains, including radiological imaging (Lebovitz et al., 2022; Jussupow et al., 2021), workflow optimization (Topol, 2019; Ahmed et al., 2022), and intelligent health management (Topol, 2019), alleviating healthcare professionals’ workload and augmenting the precision of clinical diagnosis and treatment decision-making (Ahmed et al., 2022; Delshad et al., 2021). For instance, in radiological imaging, AI systems leveraging big data techniques assist physicians in rapidly and accurately analyzing CT, X-ray, MRI, and other imaging data, automatically identifying potential lesion areas. This improves the accuracy and efficiency of detecting conditions such as lung cancer (Topol, 2019). Moreover, AI can extract pertinent information from patient histories, automatically generate medical summaries and treatment recommendations, and update medical records in real time. This streamlines health data management, enabling physicians to focus on clinical judgment while ensuring accurate and up-to-date patient information (Jussupow et al., 2021; Topol, 2019). These technological advancements not only improve the efficiency of medical services but also profoundly impact the work experiences and work well-being of healthcare professionals. As the cornerstone of the healthcare system, the work well-being of healthcare professionals is intrinsically tied to the quality of patient care, team collaboration, and the overall sustainability of the healthcare system (Bamforth et al., 2023). Given the ongoing and widespread integration of medical AI, it has become increasingly critical to conduct a comprehensive examination of its impact on healthcare professionals’ work well-being. Such an investigation is essential for optimizing the application of AI technologies and fostering sustainable work environments in healthcare settings.

A comprehensive review of the existing literature reveals that research on medical AI has primarily concentrated on examining technological acceptance among patients and healthcare professionals (Huo et al., 2024a; Huo et al., 2024b; Cai et al., 2024; Huo et al., 2023; Buck et al., 2022). Despite the rapid adoption of AI technologies in medical practice, there remains a significant gap in understanding the full impact of AI on healthcare professionals, particularly with respect to its utility and psychological implications. In other professional domains, the literature on AI usage has largely focused on its effects on work outcomes (Man Tang et al., 2022; Shao et al., 2024; Chowdhury et al., 2023; Malik et al., 2023). Existing studies have approached AI usage through theoretical lenses such as complementarity and role theory (Man Tang et al., 2022), self-regulation theory (Man Tang et al., 2023), and cognitive load theory (Shao et al., 2024), predominantly analyzing how AI usage affects job performance. However, these investigations have critically overlooked subjective work experience related to employee work well-being, thereby presenting a notable research gap. The introduction of AI transcends mere performance optimization; it fundamentally reshapes work experiences and psychological dynamics within work environments (Chowdhury et al., 2023; Malik et al., 2023). While current empirical research predominantly conceptualizes AI as a performance enhancement tool, it fails to appreciate the technology’s profound transformative potential on professionals’ work processes and experiences. The nuanced mechanisms through which AI usage influences work well-being remain largely unexplored and theoretically underdeveloped. Despite the pervasive integration of medical AI in healthcare, there is still a substantial deficit in our understanding of how, when, and to what extent AI usage affects the work well-being of healthcare professionals (Man Tang et al., 2023; Au-Yong-Oliveira et al., 2021; Bauer & Thamm, 2021).

Accordingly, this study aims to bridge the gap in existing research by examining the impact of AI usage on healthcare professionals’ work well-being. We argue that in high-stress medical environments, medical AI offers the potential to enhance work efficiency by automating routine tasks, streamlining diagnostic processes, and supporting clinical decision-making (Bekbolatova et al., 2024), thereby alleviating professional burden and improving their work well-being. To frame this exploration, we draw upon Self-Determination Theory (SDT), which offers a comprehensive theoretical lens for understanding the complex relationship between AI usage and healthcare professionals’ work well-being. According to SDT, basic psychological needs serve as key mediators linking contextual factors, such as AI adoption, to employee well-being (Ryan & Deci, 2000; Duan et al., 2024). SDT posits that three fundamental psychological needs—autonomy, competence, and relatedness—are essential for fostering optimal motivation and well-being. When work environments meet these needs, employees are more likely to experience heightened intrinsic motivation and enhanced psychological well-being (Olafsen & Frølund, 2018; Van den Broeck et al., 2016). Building on this framework, we propose that AI usage influences these three dimensions of psychological needs satisfaction in distinct ways. First, AI can enhance the needs for autonomy and competence satisfaction of healthcare professionals by improving clinical decision-making efficiency and supporting skill development, thereby boosting job performance and intrinsic motivation. Second, AI can foster the need for relatedness satisfaction by strengthening healthcare professionals’ sense of connections and communications, contributing to greater well-being and satisfaction. Together, these three basic psychological needs function synergistically to promote healthcare professionals’ work well-being.

Building upon the framework of SDT, the present study also seeks to explore the boundary conditions under which AI utilization affects psychological needs satisfaction and work well-being. The existing literature in the healthcare domain has primarily focused on the technology itself (Topol, 2019), its features (Jussupow et al., 2021), or individual traits as factors influencing psychological needs (Arslan et al., 2022). However, there is a notable gap in research addressing how unique job characteristics within the medical field may moderate these relationships. In this context, McAnally and Hagger (2024) emphasize that job characteristics can interact with contextual factors, such as AI adoption, to influence the satisfaction of basic psychological needs, which in turn affects employees’ psychological states and behaviors. Building on this insight, we propose job complexity as a critical moderating boundary condition in the relationship between the use of AI, psychological needs satisfaction, and work well-being. By considering varying levels of job complexity, this study aims to deepen our understanding of how AI impacts healthcare professionals’ work experiences across medical work environments.

**Figure 1:**
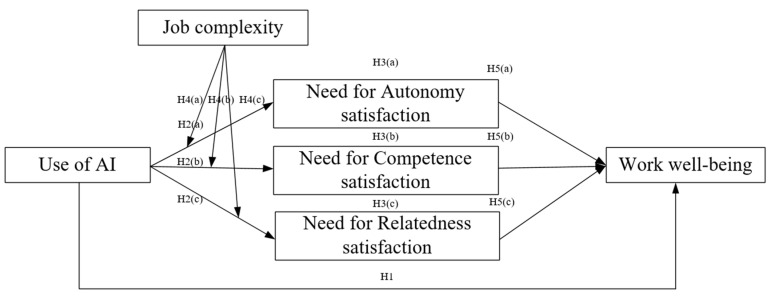
The proposed theoretical model.

In conclusion, our research aims to address a critical question: how (i.e., through psychological needs satisfaction) and when (i.e., in relation to job complexity) does medical AI usage influence healthcare professionals’ work well-being? To answer this question, we develop a comprehensive theoretical framework to elucidate the impact of medical AI on healthcare professionals’ work well-being (as illustrated in Figure 1). The model is subsequently tested using data from 280 online survey responses collected from healthcare professionals in Chinese hospitals. Our study makes several key contributions. First, by drawing on SDT, we shift the research focus beyond technological acceptance to explore the positive and nuanced psychological impacts of AI usage (Huo et al., 2024b; Huo et al., 2023). By examining healthcare professionals’ subjective experiences following the implementation of AI technologies, we move beyond traditional performance-centered frameworks that primarily focus on task performance (Man Tang et al., 2022, 2023; Shao et al., 2024; Leroy, 2024). Second, we extend the application of SDT by incorporating the need for autonomy satisfaction, need for competence satisfaction, and need for relatedness satisfaction into the AI workplace context. This approach offers a theoretically grounded explanation of how AI usage influences work well-being, thereby expanding the theoretical boundaries of SDT within digital work environments. Finally, we investigate the boundary conditions of use of AI by introducing job complexity as a critical moderating variable. While the existing healthcare literature predominantly focuses on technological features (Jussupow et al., 2021; Topol, 2019; Xu et al., 2023), our study addresses a significant research gap by examining the role of job characteristics as a moderating factor. By considering the unique work characteristics of the medical field, we provide a more nuanced understanding of the psychological implications of AI usage in healthcare settings.

## 2. Theoretical Background and Hypotheses Development

### 2.1. Use of AI and Work Well-Being

***Self-Determination Theory***. Self-Determination Theory (SDT), introduced in the late 1970s by American psychologists Edward Deci and Richard Ryan, explores the dynamics of motivation in human behavior. SDT elucidates how external environmental factors influence internal motivation and the process of internalization, shedding light on the pathways that shape individual motivation (Ryan & Deci, 2000; Deci & Ryan, 1985; Ryan & Deci, 2020; Van den Broeck et al., 2016). Empirical research within the SDT framework, viewed through an interdisciplinary lens, consistently demonstrates that when individuals receive support from the social environment and internal demands for autonomy, competence, and relatedness, it not only fosters high-quality motivation but also enhances health-promoting behaviors, vitality, and the pursuit of life goals, thereby contributing to overall well-being (Deci & Ryan, 1985; Ryan & Deci, 2020; Ryan et al., 2022; Sheldon & Prentice, 2019; Gillison et al., 2019). As a result, an increasing body of literature suggests that SDT provides a robust and comprehensive framework for understanding human motivation, particularly in the context of a rapidly changing world. It offers valuable insights into the underlying mechanisms of behavior change, both in real-time and in anticipation of future shifts. Central to this theory are three dimensions of satisfaction of psychological needs—autonomy, competence, and relatedness—that foster internal motivation. Autonomy emphasizes self-determination and self-regulation, reflecting a sense of ownership and psychological freedom in one’s actions. Competence pertains to the need to proficiently interact with the environment and cultivate new skills, capturing an individual’s innate drive to explore, manipulate their surroundings, and overcome challenges. Relatedness represents the fundamental need for social connection, including the feeling of being close to and valued by others, underscoring its essential role in emotional and social well-being.

Building on SDT, AI technologies in the healthcare sector play a pivotal role as facilitators within the social work environment, shaping healthcare professionals’ motivation and enhancing the outcomes of their daily tasks. In the management of thyroid nodules, AI-assisted tools effectively alleviate physicians’ workload by significantly reducing the time required for image review. These tools offer notable benefits in improving diagnostic efficiency while maintaining high levels of diagnostic accuracy (Chiniara & Bentein, 2016; Moor et al., 2023). As AI technologies continue to evolve, healthcare professionals are increasingly utilizing AI tools to optimize patient care (Topol, 2019; Moor et al., 2023). For example, AI-assisted CT tools are employed by radiologists as supplementary aids following their initial independent assessments, thereby improving diagnostic efficiency, particularly in cancer detection, such as lung cancer. These tools not only provide reliable foundations for confirming diagnoses but also expedite the evaluation of disease progression and the development of treatment plans.

On the one hand, medical AI usage alleviates the daily burden of strenuous tasks for healthcare professionals, allowing them to dedicate more time and energy to complex, high-value responsibilities. On the other hand, by validating their initial judgments with AI-generated results such as CT-image results, healthcare professionals can enhance their competence and expand their knowledge. This dynamic interaction between AI tools and healthcare professionals can also support the satisfaction of intrinsic motivation and need, helping individuals align their competencies with the demands of an evolving technological work environment. Moreover, intrinsically motivated behaviors, which are more closely aligned with an individual’s core values and interests, tend to result in higher levels of satisfaction and sustained well-being. Extensive research has demonstrated that intrinsic motivation is not only linked to short-term enjoyment but also contributes to long-term mental health and work well-being (Van den Broeck et al., 2016; Ryan & Deci, 2020; Ryan et al., 2022; Sheldon & Prentice, 2019). Consequently, this alignment between AI-driven tasks and intrinsic motivation may ultimately foster improved job satisfaction and overall work well-being (Ryan & Deci, 2020; Wallace et al., 2016).

Therefore, we propose the following hypothesis:

**H1.**  *Healthcare professionals’ use of AI is positively associated with work well-being.*

### 2.2. Use of AI and Psychological Needs Satisfaction

Although a substantial body of literature highlights the “mixed blessings and disadvantages” associated with the integration of AI and human professionals (Topol, 2019; Jia et al., 2024; Yam et al., 2023; Liang et al., 2022; Teng et al., 2024; Ding, 2021), the introduction of AI in healthcare organizations, such as hospitals, underscores a growing consensus among academics regarding the advantages of integrating intelligent technologies into the workplace (Man Tang et al., 2023). For example, while Topol (2019) provides an overview of the current state of medical AI development and cites various concerns, including data privacy risks (such as the increased likelihood of identifying individuals through genomic sequences in vast databases, exacerbated by hacking and data breaches) (Albahri et al., 2023), algorithmic bias (e.g., diagnostic algorithms for melanoma that fail to account for skin color, or genomic databases that remain grossly under-representative of minorities) (Chen et al., 2023), and lack of transparency (where the opacity of AI systems creates uncertainty among professionals when AI outputs deviate from clinical judgments without clear explanations, requiring additional time and effort to verify results, which may decrease productivity) (Jussupow et al., 2021). However, Topol (2019) and numerous researchers argue that, despite these challenges, nearly all types of clinicians, from specialists to caregivers, will increasingly adopt AI technologies to address critical issues such as healthcare resource imbalances and the shortage of healthcare professionals (Song et al., 2024; Feng & Hua, 2022; Zhang & Zhao, 2024; Li & Qin, 2023; Gu et al., 2019; Fan et al., 2020; Pan et al., 2019).

Based on the aforementioned information, AI serves as an external environmental stimulus that has catalyzed the redesign and optimization of healthcare workflows (Buck et al., 2022), empowering healthcare professionals to make more autonomous decisions by leveraging AI for diagnosis, treatment planning, and patient management (Man Tang et al., 2023; Makarius et al., 2020; Zahlan et al., 2023). For instance, the Galen image-recognition platform’s AI-driven algorithms could assess and classify cancers, thereby enhancing the diagnostic accuracy of pathologists while also reducing the time required for diagnosis. Similarly, See-Mode, a Singapore-based company, integrates medical imaging with AI to assist clinicians in predicting strokes, potentially saving lives (Zahlan et al., 2023). The adoption of such AI technologies largely depends on the individual clinician’s willingness, positioning them as supplementary tools to support healthcare professionals. Importantly, these AI-powered devices are not confined to specific medical specialties; rather, they offer broad benefits across various sectors of healthcare, supporting healthcare professionals in diverse contexts to varying extents (Khan et al., 2024). According to SDT, theorists suggest that individuals possess intrinsic tendencies toward integration, growth, and well-being, contingent upon the satisfaction of basic psychological needs. In this context, AI usage enables healthcare professionals to allocate more time and resources toward personal and professional development, including the personalized management of complex, non-procedural cases and the exploration of innovative treatment options. Thus, AI-assisted applications allow physicians to achieve greater autonomy in their work, thereby partially satisfying their need for autonomy in the process.

The utilization of intelligent machines for clinical decision support represents a transformative approach in healthcare. For instance, advanced healthcare AI models can diagnose patients by analyzing data from digitized electronic health records (EHRs), summarizing the patient’s current status, predicting potential future developments, and recommending treatment plans to assist physicians in their diagnoses (Kalra et al., 2024; Spring et al., 2022; Youssef et al., 2023). In this integrated interaction between human experts and machines, both parties leverage their complementary strengths. Typically, machine outputs are used to challenge initial human judgments, while human inputs serve to refine and optimize machine-generated outputs. This synergistic relationship facilitates knowledge translation and integration, enabling both humans and machines to learn from each other’s inputs and outputs, thereby enhancing their respective capabilities (Jussupow et al., 2021; Moor et al., 2023). Specifically, numerous AI-assisted diagnostic tools incorporate built-in feedback mechanisms that enable physicians to provide input after reviewing and correcting the AI-generated diagnostic results. For instance, in early breast cancer screening, an AI system is trained on a large dataset of medical images to automatically detect potential lesion areas and generate preliminary diagnostic outputs. However, the AI system’s output is not infallible and may include occasional false positives or missed diagnoses. In such instances, physicians can manually annotate the correct lesion areas and specify the nature of the misdiagnosis through the system’s interface. These annotations are then stored and incorporated into the system’s training dataset, contributing to the refinement of the model. By continuously accumulating feedback data from clinicians, the AI system can progressively enhance its diagnostic accuracy over time. Through this process, human experts can expand and deepen their expertise, while machines enhance their accuracy and efficiency. This collaboration not only improves overall performance but also fosters innovation and continuous learning. Consequently, as individuals engage with new technologies, they partially satisfy their needs to control and navigate their environment by anticipating the challenges posed by these technologies and acquiring proficiency in the novel techniques required for their work.

AI acts as a technological enabler, facilitating interaction with smart devices and fostering new forms of communication within healthcare. On the one hand, the integration of AI in clinical data sharing has promoted cross-sector collaboration, reinforcing its role in the medical field. These technologies allow healthcare professionals to collaborate more effectively, monitor treatment efficacy, and adjust strategies in real time (Man Tang et al., 2023; Youssef et al., 2023). On the other hand, the impact of technological advancements on individuals is increasingly complex (Wang et al., 2023). The shift from viewing machines as mere tools for production to recognizing them as integral components of organizational and economic systems carries significant implications (Arslan et al., 2022). Healthcare professionals may perceive AI technology either as a simple tool that supports their tasks or as a more emotionally significant entity. When AI is considered a team member, the collaborative process not only aids in task completion but also enhances expertise through interactive engagement. This iterative collaboration with AI-assisted diagnostic tools—characterized by continuous feedback and input–output exchanges, as previously discussed—mirrors the dynamics of human team communication. Such interactions enable healthcare professionals to experience a sense of connection and value, akin to the mutual recognition found in human collaboration. As a result, this AI-based model strengthens communication and mutual support among team members, thereby enhancing physicians’ need for relatedness satisfaction—a sense of connection and belonging with others (Arslan et al., 2022).

Taken together, we propose the following hypothesis:

**H2.**  *The use of AI is positively associated with the need for (a) autonomy, (b) competence, and (c) relatedness satisfaction of healthcare professionals.*

### 2.3. The Mediation of Three Dimensions of Psychological Needs Satisfaction

Within the framework of SDT and healthcare AI, it becomes evident that the integration of AI not only improves the efficiency and quality of patient care but also addresses the fundamental psychological needs of healthcare professionals, specifically the need for autonomy satisfaction, need for competence satisfaction, and need for relatedness satisfaction. The satisfaction of these needs plays a critical role in promoting physicians’ mental health, motivation, and overall well-being (Brady et al., 2020). Extending this theoretical framework to healthcare AI, we observe that AI systems facilitate the gratification of these psychological needs, which, in turn, can enhance physicians’ work well-being, thereby increasing their motivation and job performance (Van den Broeck et al., 2016; Kahn, 1990).

As AI optimizes workflows, it enables healthcare professionals to exercise greater autonomy in decision-making processes related to diagnosis, treatment planning, and patient management. This enhanced autonomy fosters a heightened sense of control over their work while also reducing external pressures (Cramarenco et al., 2023). As a result, the satisfaction of autonomy not only bolsters intrinsic motivation but also encourages more active engagement in professional activities (Van den Broeck et al., 2016; Hood & Patton, 2022). Over time, interactions with AI allow healthcare professionals to refine their skills and expand their expertise, further reinforcing their sense of competence. Together, the satisfaction of autonomy and competence needs contributes to a stronger sense of agency within their professional environment, fostering empowerment both at work and in life and promoting work well-being.

In addition, according to SDT, the need for relatedness satisfaction is intricately linked to an individual’s mental health. In the healthcare context, when individuals experience meaningful connections and mutual concern during their interactions with AI, they gain a more profound perspective on life, leading to enhanced well-being and satisfaction. Extensive literature supports the view that the satisfaction of these basic psychological needs significantly contributes to improved health and well-being (Van den Broeck et al., 2016). Based on these insights, we propose the following hypothesis:

**H3.**  *The needs for (a) autonomy, (b) competence, and (c) relatedness mediate the relationship between the use of AI and healthcare professionals’ well-being at work.*

### 2.4. The Moderating Impact of Job Complexity

The preceding arguments have highlighted the positive impact of AI utilization on the performance of healthcare professionals. However, in the healthcare sector—where innovation and rigor are intertwined—it is reasonable to posit that the user-centered use of AI may diversely impact personnel. Integrating Cognitive Evaluation Theory (CET), a sub-theory of SDT, helps to explain how intrinsic motivation can vary depending on environmental conditions. According to CET, intrinsic motivation flourishes when individuals have the opportunity to pursue their personal interests, goals, and values within a supportive environment. Conversely, when environmental factors constrain these pursuits, or when individuals are subject to extrinsic rewards or punishments for engaging in controlling behaviors, intrinsic motivation is likely to diminish (Ryan & Deci, 2000). Consequently, this perspective reinforces the notion that psychological needs are significantly influenced by the surrounding work environment, including factors such as task characteristics (e.g., complexity and variety) (Parent-Rocheleau & Parker, 2022).

Job complexity (JC), a key attribute of job characteristics, refers to the depth and breadth of psychological demands faced by employees in the workplace (Morgeson & Humphrey, 2006). Generally, higher levels of job complexity are associated with increased mental demands and challenges, which can lead to positive motivational outcomes (Humphrey et al., 2007; Parent-Rocheleau & Parker, 2022). Fasbender and Gerpott (2023) emphasize that job characteristics such as job complexity, responsibility, and autonomy have greater motivational potential than more routinized and formalized job attributes.

In the healthcare context, however, we contend that as job complexity increases, tasks become more detailed and specialized, thereby intensifying the demands placed on healthcare professionals’ expertise. High-complexity tasks, driven by the intrinsic motivation to provide accurate patient care, often necessitate more in-depth analysis and judgment from physicians. In such situations, healthcare AI systems are generally limited in their ability to address these complexities (Lebovitz et al., 2022). The findings of Jussupow et al. (2021) further support this view, as demonstrated by their 10-month qualitative study conducted across three departments (Breast Imaging, Chest Imaging, and Pediatric Imaging) at a teaching hospital in the United States. The study revealed that, particularly in the Breast Imaging and Pediatric Imaging departments, physicians frequently encounter significant uncertainties in disease diagnosis, especially when confirming cancer diagnoses. These uncertainties, in turn, limit the effectiveness of AI in these clinical contexts. For example, in breast cancer diagnosis, the high incidence and risk profile of the disease render early detection of the most treatable stages critical. Any errors in assessment carry substantial risks, potentially leading to significant negative impacts on patient outcomes. Moreover, the variability in patient anatomy and the inherent complexity of breast tissue contribute to the characterization of breast cancer as a multifaceted and unpredictable disease. In practice, radiologists often begin their assessments by reviewing mammographic images, a process fraught with uncertainty as they attempt to identify abnormal regions within the complex architecture of breast tissue and to determine the likelihood of malignancy or benignity.

Consequently, as the job complexity increases, the accuracy and reliability of medical AI may decline. This is because complex tasks often involve greater variability and uncertainty, which can adversely affect the predictions and judgments made by AI systems (Topol, 2019; Wang et al., 2024). In such scenarios, the assistance provided by AI may prove insufficient to address the diverse and intricate challenges healthcare professionals encounter, thus failing to meet all professional requirements. When faced with a highly complex job, healthcare professionals may derive limited benefits from the use of AI, thereby weakening the relationship between the use of AI and psychological needs satisfaction. Therefore, we propose the following hypotheses:

**H4a.**  *Job complexity moderates the impact of AI usage on the need for autonomy satisfaction, such that the relationship is weaker with a higher level of job complexity.*

**H4b.**  *Job complexity moderates the impact of AI usage on the need for competence satisfaction, such that the relationship is weaker with a higher level of job complexity.*

**H4c.**  *Job complexity moderates the impact of AI usage on the need for relatedness satisfaction, such that the relationship is weaker with a higher level of job complexity.*

Building on the preceding arguments, we hypothesize that the use of AI leads to an increase in the satisfaction of the psychological needs for autonomy, competence, and relatedness (H2). Taking it one step further, we posit that the need for autonomy, competence, and relatedness satisfaction serves as a mediator in the positive and indirect relationship between the use of AI and work well-being (H3). In addition, we propose that job complexity can moderate the relationship between the use of AI and the need for autonomy, competence and relatedness satisfaction (H4a, H4b, and H4c). These relationships can be conceptualized within a moderated mediation model. Therefore, combining H1–H4, we propose the following moderated mediation hypotheses:

**H5a.**  *Job complexity moderates the mediation effect of the need for autonomy satisfaction on the relationship between AI usage and work well-being, such that this mediation effect will be weaker when job complexity is higher.*

**H5b.**  *Job complexity moderates the mediation effect of the need for competence satisfaction on the relationship between AI usage and work well-being, such that this mediation effect will be weaker when job complexity is higher.*

**H5c.**  *Job complexity moderates the mediation effect of the need for relatedness satisfaction on the relationship between AI usage and work well-being, such that this mediation effect will be weaker when job complexity is higher.*

## 3. Methods

### 3.1. Design, Setting, and Participants

To test our hypotheses and mitigate the potential impact of common method bias (CMB)^1^ in the collection of self-reported data, we conducted two separate questionnaire surveys distributed via online platforms in China. Given that our study aims at empirical validation (focuses on the extent of the use of AI rather than the mere use of AI itself), all participants, including healthcare professionals from various departments such as radiology, ophthalmology, cardiology, and others, were required to have prior experience with medical AI (Huo et al., 2024a). The first wave of data was collected in February 2024, during which demographic information (i.e., gender, age, educational background, and job tenure) was gathered, along with measures assessing the use of AI and job complexity. The second wave of data collection occurred in March 2024, focusing on the constructs of psychological needs satisfaction (i.e., autonomy, competence, and relatedness) and work well-being.

After matching the data from the two waves and excluding incomplete responses as well as those failing to meet the screening criteria, a total of 280 valid responses were retained. Among the participants, 29.3% were male, 42.9% were aged between 26 and 35 years, and 55% had more than six years of work experience. Regarding educational background, 41.1% held a bachelor’s degree, while 21.4% held a master’s degree. These demographic characteristics may influence the relationships between the study variables in different ways. For instance, younger healthcare professionals may exhibit greater enthusiasm and openness toward adopting AI in the workplace, whereas those with higher levels of education may possess a more advanced understanding of AI technology, potentially leading to more favorable attitudes toward its use (Bühler et al., 2022). The potential impact of these demographic factors will be addressed in the subsequent analysis.

### 3.2. Variables and Measurement

In addition to collecting demographic data, we measured all other constructs in our survey using a five-point Likert scale (for its simplicity, ease of understanding, and convenience for quantitative analysis), ranging from 1 (strongly disagree) to 5 (strongly agree) (Chyung et al., 2017). The original English scales were translated into Chinese using a back-translation process, conducted by a team of researchers proficient in both Chinese and English to ensure translation accuracy. Additionally, to maintain the cultural appropriateness of the scale, we engaged in multiple discussions and revisions during the translation process, particularly addressing any ambiguous items in the original scale through detailed discussions and adjustments. To ensure that the translation preserved the core meaning of the original items while aligning with the cultural background and understanding of the research participants, we conducted a pilot survey with experts in the fields of medical AI and organizational behavior before the formal data collection. After repeatedly confirming the clarity of the measurements and resolving any ambiguities, we finalized the formal survey.

***Use of AI.*** A three-item scale, adopted from Man Tang et al. (2023), was used to measure the extent to which healthcare professionals in hospitals use medical AI. An example of items is “I depend on medical AI to help me with work-related tasks”, and the Cronbach’s alpha coefficient for this construct was 0.878 (as shown in Table 1).

***Psychological needs satisfaction.*** We measured the construct based on La Guardia et al.’s (2000) 9-item scale for the implementation of medical AI in medical settings. Needs satisfaction is divided into three dimensions, including need for autonomy satisfaction, need for competence satisfaction, and need for relatedness satisfaction, with three measure items for each dimension. For the need for autonomy satisfaction, an illustrative item is “When I collaborate with medical AI, I can still follow my own approach to diagnosis and treatment”. For the need for competence satisfaction, a demonstrative example is “When I collaborate with medical AI, I feel capable in my work”. As for the need for relatedness satisfaction, a sample item is “When collaborating with medical AI, I feel as if it cares for and supports me like a colleague”. The Cronbach’s alpha values for these dimensions were 0.755, 0.784, and 0.803, respectively.

***Work well-being.*** To assess healthcare professionals’ work well-being, we utilized a situationally adapted six-item scale developed by Zheng et al. (2015). A representative item from this scale is “Since the introduction of medical AI, I find my work to be more interesting”. The Cronbach’s alpha of this variable was 0.888.

***Job complexity.*** Job complexity in healthcare institutions was assessed using a four-item scale adapted from Zacher et al. (2010). An example item is “My current work tasks are very complex”. The scale demonstrated good internal consistency, with a Cronbach’s alpha of 0.860. Additionally, the Cronbach’s alpha for all other measured variables ranged from 0.755 to 0.860, with all exceeding the acceptable threshold of 0.7, thereby indicating strong internal consistency across the variables.

***Control variables.*** Gender, age, education, and job tenure were included as control variables due to their potential impact on the study outcomes. Gender may influence healthcare professionals’ acceptance of AI, while age is related to career experience and technological adaptability. Education reflects the ability to comprehend and apply technological tools, and tenure of job in the workforce may affect proficiency with technology. Controlling for these variables helps minimize confounding effects and ensures the robustness of the study’s findings (Bühler et al., 2022).

## 4. Analyses and Results

### 4.1. Reliability and Confirmatory Factor Analysis

Utilizing confirmatory factor analysis (CFA), we extracted the factor loadings for the items in this study. Based on these loadings, we calculated the composite reliability (CR) and average variance extracted (AVE) for the six variables. All constructs exhibited Cronbach’s α and CR values greater than 0.7, while the AVE values exceeded 0.5, indicating strong structural validity. These results demonstrate good internal consistency within the measurement model, ensuring the accuracy and reliability of our study findings (Fornell & Larcker, 1981).

### 4.2. Common Method Bias and Discriminant Validity

To verify the absence of underlying methodological factors, specifically the presence of serious CMB, we first conducted Harman’s one-factor test. The results indicated that the predominant factor accounted for only 36.88% of the variance, falling below the 50% threshold recommended by Fuller et al. (2016).

Considering the possible limitations of Harman’s one-factor test, we adopted the CFA Marker Technique suggested by Williams et al. (2010) to further test for CMB. First, we selected age as an unrelated marker variable, which is also not significantly correlated with the six substantive latent variables (use of AI, need for autonomy satisfaction, need for competence satisfaction, need for relatedness satisfaction, work well-being, and job complexity) in our research model (in Table 2). The presence of CMB was assessed by comparing the chi-square tests across several models. Specifically, in the CFA model, we allowed the six substantive latent variables to be fully correlated with the marker variable to estimate the factor loadings and measurement error variances of the marker variable. Next, we constructed a baseline model in which the marker variable was assumed to be orthogonal to the other latent variables, with its factor loadings and error variances fixed. Additionally, we compared the method-C and method-U models, where the former assumes equal measurement effects for all substantive indicators, while the latter allows for different effects. Finally, we use the factor correlations obtained from the baseline model for substantive variables as fixed values in the method-U model to assess the method-R model. As presented in Table 3, a significant differences that is observed between the method-C model and the baseline model [$\Delta \chi^{2}\left(\Delta df=1\right)=0.53,\;p<0.05$]. Further, we compared the significant differences between the baseline and method-U models. The evaluation of the method-U model and its comparison with the baseline model revealed a significant difference [$\Delta \chi^{2}\left(\Delta df=22\right)=35.325,\;\;p<0.05$]. Consequently, additional testing was performed. A chi-square difference test between the method-U and method-R models showed no significant difference between the two models [$\Delta \chi^{2}\left(\Delta df=16\right)=10.456,\;\;p>0.05$]. Thus, the CFA Marker Technique indicated that there was no serious CMB in our research model.

As shown in Table 4, the six-factor model demonstrates a good fit ($\chi^{2}/df$ = 1.534 < 3; RMSEA = 0.044 < 0.05; SRMR = 0.0488 < 0.05; TLI = 0.962 > 0.9; CFI = 0.968 > 0.9). Additionally, compared to the other models (ranging from the one-factor model to the five-factor model), the six-factor model showed the best fit, indicating that its discriminant validity was acceptable.

### 4.3. Descriptive Statistics Analysis

Table 2 presents the descriptive statistics of the variables, including their means, standard deviations, and inter-construct correlations. The results indicated that there were no significant relationships between the control variables (gender, age, education, and tenure) and the dependent variable, work well-being. Therefore, these control variables were unlikely to influence the relationships between the other variables in the research model and work well-being. Furthermore, we found significant positive correlations between the use of AI and the autonomy (r = 0.256, *p* < 0.01), competence (r = 0.324, *p* < 0.01), and relatedness (r = 0.33, *p* < 0.01) of the three dimensions of psychological needs satisfaction. Likewise, these three dimensions were significantly correlated with work well-being. These findings establish a basis for the subsequent hypothesis testing in this study.

### 4.4. Hypothesis Testing

We further conducted hierarchical regression analysis on the variables presented in Table 5. In Model 2, after including both the independent and control variables, we observed a significant positive regression coefficient for the effect of the use of AI on work well-being (β = 0.297, *p* < 0.001), thus supporting H1. Additionally, Model 4 revealed a significant positive relationship between the use of AI and the need for autonomy satisfaction (β = 0.269, *p* < 0.001), confirming H2a. Similarly, Models 8 and 12 demonstrated significant positive relationships between the use of AI and the needs for competence (β = 0.332, *p* < 0.001) and relatedness (β = 0.358, *p* < 0.001) satisfaction, thereby supporting both H2b and H2c.

#### 4.4.1. Mediation Effects: Three Dimensions of Psychological Needs Satisfaction

**Figure 2:**
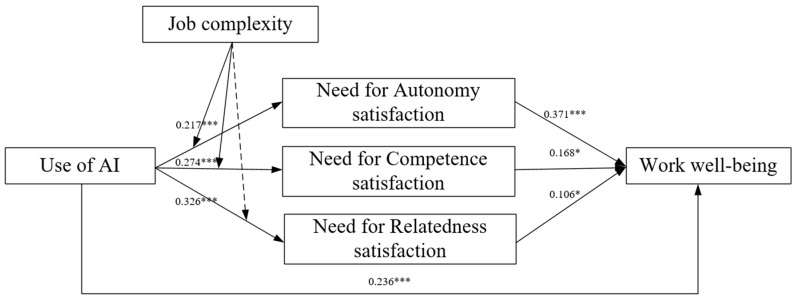
Results of model effect analysis. Note: * *p* < 0.05. *** *p* < 0.001.

The bootstrap method was used to confirm the statistical significance of the path coefficients and the mediation effect through 5000 bootstrap samples. Hypotheses were considered supported if 0 was not included within the 95% confidence interval (CI) (Wang et al., 2023). As shown in Table 6, the total effect of the use of AI on work well-being in our research model is statistically significant (β = 0.236, *p* < 0.01), thus providing further support for H1. Additionally, the three dimensions of psychological needs satisfaction—autonomy (β = 0.081, 95% CI [0.033, 0.138]), competence (β = 0.046, 95% CI [0.002, 0.103]), and relatedness (β = 0.035, 95% CI [0.003, 0.073])—were found to significantly mediate the relationship between the use of AI and work well-being (see Table 6 and Figure 2). These results support H3a, H3b, and H3c. Moreover, it is noteworthy that the three dimensions of basic psychological needs fully mediate the relationship between the use of AI and work well-being, as indicated in Table 6.

#### 4.4.2. The Moderation Effect of Job Complexity

To test H4a, H4b, and H4c, we employed Process Model 7 in SPSS 26.0 to examine the moderating effect of job complexity on the relationship between the use of AI and psychological needs satisfaction (need for autonomy satisfaction, need for competence satisfaction, and need for relatedness satisfaction). First, as indicated in Model 6 of Table 5, the interaction term between the use of AI and job complexity exhibits a significant negative effect on the need for autonomy satisfaction (β = −0.13, *p* < 0.05). The Johnson–Neyman test further revealed that the relationship between the use of AI and the need for autonomy satisfaction became non-significant (*p* > 0.05) when job complexity exceeded a value of 3.778, suggesting that higher levels of job complexity weaken the relationship between the use of AI and the need for autonomy satisfaction. Thus, the results support H4a. Second, as shown in Model 10 of Table 5, there is also a significant negative effect of the interaction term between the use of AI and job complexity on the need for competence satisfaction (β = −0.146, *p* < 0.05). Similarly, the Johnson–Neyman test indicated that when the value of job complexity exceeded 4.197, the effect of the use of AI on the need for competence satisfaction was non-significant (*p* > 0.05), further demonstrating that increased job complexity weakens the relationship between these constructs. Consequently, H4b is supported.

**Figure 3:**
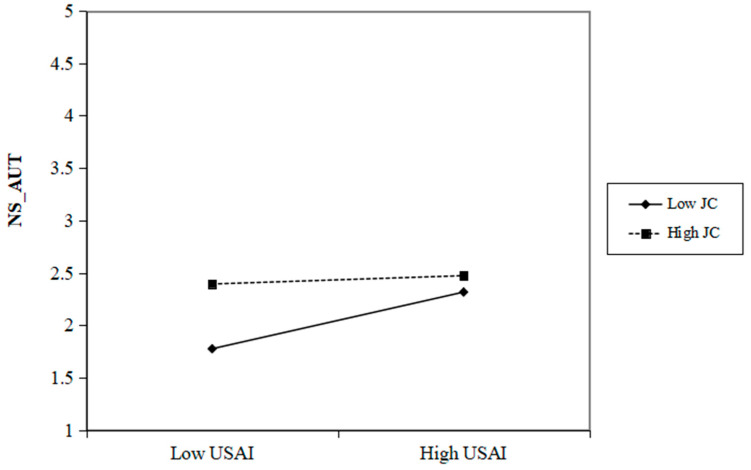
Two-way interaction between USAI and JC for prediction NS_AUT. Note: USAI=Use of AI. NS_AUT = Need for Autonomy Satisfaction. JC = Job Complexity.

**Figure 4:**
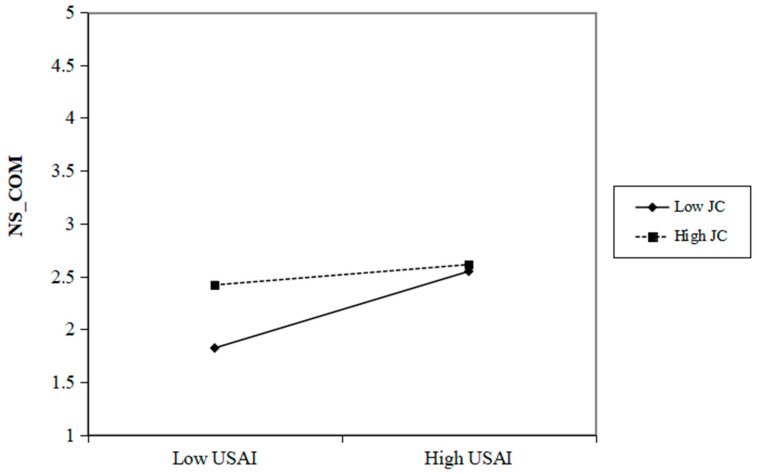
Two-way interaction between USAI and JC for prediction NS_COM. Note: USAI=Use of AI. NS_COM = Need for Competence satisfaction. JC = Job Complexity.

Additionally, the moderating influence of job complexity on the relationships between the use of AI and the needs for autonomy and competence satisfaction are illustrated in Figure 3 and Figure 4. However, as observed in Model 14 (in Table 5), the interaction term between the use of AI and job complexity does not yield a significant effect on the need for relatedness satisfaction (β = −0.025, *p* > 0.05). Consequently, H4c is not supported.

#### 4.4.3. Moderated Mediation Effect Test

To further verify the existence of the moderated mediation effect, we employed the methodology recommended by James and Brett (1984). Based on the results from the moderating effects tested in H4a, H4b, and H4c, we found that job complexity significantly moderates the relationship between the use of AI and healthcare professionals’ need for autonomy satisfaction. Further, to test hypotheses H5a, H5b, and H5c, we conducted a moderated mediation analysis using Process Model 7 in SPSS 26.0, with 5000 bootstrap samples at the 95% confidence interval. As shown in Table 7, for this path of need for autonomy satisfaction as a mediator, job complexity as the moderator, the Index is −0.035, with a Boot standard error of 0.019, and the confidence interval (BootCI) at the 95% level is [−0.077, −0.002], which does not contain 0, indicating that the moderated mediation effect of the pathway “use of AI—need for autonomy satisfaction—work well-being” is significant. These findings validate the moderated mediation role of job complexity in the relationship between the use of AI and work well-being, thereby supporting H5a.

Furthermore, for this pathway in which the need for competence satisfaction served as a mediator (Table 7), Index = −0.018, with a Boot standard error of 0.013, and BootCI at the 95% level is [−0.051, 0], which contains 0. This suggests that the moderated mediation effect of job complexity in the pathway “use of AI—need for competence satisfaction-work well-being” is not significant. Therefore, the moderated mediating effect of job complexity impacting the mediating role of the need for competence satisfaction on the relationship between the use of AI and work well-being is not validated, and H5b is not supported.

In the same way, Table 7 presents the role of job complexity for the pathway of “use of AI —need for relatedness satisfaction— work well-being”, which indicates the moderated mediation effect is not significant (Index = −0.002, Boot standard error = 0.005, and BootCI at the 95% confidence level is [−0.015, 0.008]). Therefore, H5c is not supported.

## 5. Discussion

We propose a theoretical model from the lens of Self-Determined Theory to elucidate the relationship between healthcare professionals’ use of AI and their work well-being. Specifically, we argue that the three dimensions of psychological needs satisfaction (autonomy, competence, and relatedness) mediate the relationship between the AI usage and work well-being. While the existing literature highlights the “double-edged sword” of AI usage (Man Tang et al., 2023; Liang et al., 2022; Ding, 2021), our model emphasizes the potential positive outcomes of AI integration, particularly its ability to enhance healthcare professionals’ work well-being. Notably, the use of AI in healthcare can stimulate intrinsic motivation, fostering professional growth, increasing job satisfaction, and enhancing overall well-being and innovative performance (Jussupow et al., 2021; Van den Broeck et al., 2016; Gagné et al., 2022).

Contrary to findings from previous studies, our research demonstrates that job complexity acts as a boundary condition, weakening the relationship between healthcare professionals’ use of AI technologies and their satisfaction of autonomy and competence needs. This is primarily due to the challenges posed by complex medical tasks that may exceed the processing capabilities of medical AI, thereby limiting its utility in providing decision support (Topol, 2019; Shen et al., 2019). Additionally, job complexity weakens the connection between the use of AI and healthcare professionals’ sense of ownership at work, which in turn attenuates the indirect effect of AI on work well-being. Interestingly, while job complexity diminishes the link between the use of AI and the need for competence satisfaction, it does not significantly reduce the relationship between AI usage and work well-being. Healthcare professionals may experience temporary disruptions in their competence needs due to the increased effort required to resolve complex medical cases. However, their intrinsic motivation, coping strategies, and professional responsibility enable them to maintain a positive attitude and continue prioritizing patient care, ultimately sustaining their work well-being (Chan et al., 2020; Selvachandran et al., 2023; Sendak et al., 2020).

In light of these findings, while job complexity weakens the relationship between the use of AI and the need for relatedness satisfaction, healthcare professionals’ recognition of the benefits of AI adoption remains largely unaffected. Consequently, H4c and H5c are not supported. This further highlights the key findings of our study: within the context of healthcare development in China, the introduction of AI benefits healthcare professionals, AI-based devices, and other stakeholders. Moreover, our research aligns with the optimistic perspectives on AI in healthcare, as articulated by proponents such as Topol (2019), reaffirming the potential positive impact of AI in this field. We hope that these findings can inform stakeholders on the effective development and integration of AI technologies, ultimately improving healthcare outcomes and enhancing the work experiences of healthcare professionals.

### 5.1. Theoretical Implications

The work well-being of healthcare professionals is integral to both patient care and the advancement of AI in healthcare. As outlined above, our study offers significant insights into the positive performance and progression of healthcare professionals within this domain. By employing SDT as a foundational framework, we underscore the significance of intrinsic motivation, highlighting that individuals are often driven by self-transcendent values that prioritize the welfare of others over self-interest. Our findings also foster optimism regarding the integration of intelligent machines in healthcare settings, suggesting that both the creators and users of healthcare AI can recognize its potential performance benefits. Specifically, as noted earlier, the close interaction and coupling between human experts and intelligent machines facilitates the transfer and integration of knowledge, allowing both parties to learn from each other’s inputs and outputs, thereby enhancing their respective capabilities. This provides positive empirical evidence of the beneficial effects of such collaboration. This perspective can serve as a guiding principle for stakeholders in the effective development and integration of AI technologies in healthcare (Man Tang et al., 2023; Dediu et al., 2018; Economou-Zavlanos et al., 2024).

Second, our contribution lies in the articulation of an intrinsic mechanism that elucidates the relationship between the use of AI and the well-being of healthcare professionals. By incorporating the satisfaction of the psychological needs for autonomy, competence, and relatedness as mediating variables, we develop a theoretical model that interprets how employees’ adoption of smart machines in healthcare correlates with enhanced work well-being. Notably, there is a paucity of research exploring the impact of AI usage on psychological needs satisfaction within the medical field; our study addresses this gap and contributes to the understanding of the mechanisms underlying psychological needs satisfaction. Moreover, by examining the technological implications of intelligent machines and their role in satisfying healthcare professionals’ psychological needs, we provide new insights into the positive effects of human–machine collaboration in healthcare. Our findings further demonstrate that healthcare professionals can be relieved from routine, standardized tasks, allowing them to engage in more nuanced and valuable responsibilities. This shift not only facilitates the integration of human expertise with AI but also fosters the mutual advancement of both human and AI capabilities.

Third, we acknowledge that the unique characteristics of the healthcare industry establish a boundary condition for our findings, particularly regarding the limitations of job complexity on the positive aspects of healthcare AI applications. Our research highlights the detrimental effect of job complexity on the relationship between the use of AI and the satisfaction of psychological needs, thereby enhancing our understanding of the constraints associated with healthcare AI in practical settings. These limitations extend beyond technological capabilities, highlighting the inability of AI systems to fully replace healthcare professionals in making independent decisions and adapting to the diverse and complex healthcare environments. This underscores the indispensable role of healthcare professionals in medical AI applications and their irreplaceability in delivering high-quality healthcare services. Consequently, we advocate for the development of a more collaborative working model in the application of medical AI, in which AI systems serve as auxiliary tools that provide informational support to healthcare professionals. In this model, healthcare professionals would leverage their professional knowledge and experience to interpret and evaluate the insights generated by AI, facilitating a partnership that ensures the delivery of optimal patient care.

### 5.2. Practical Implications

Our research reveals several practical implications. First, we emphasize that the implementation of smart machines in healthcare settings can stimulate the intrinsic motivation of healthcare professionals, thereby enhancing their well-being at work. To maximize the benefits of AI, healthcare professionals should actively promote collaborative working models that position smart technologies as supportive tools rather than replacements in the decision-making process of healthcare professionals. Additionally, we recommend that hospital administrators highlight the advantages of user-friendly AI systems to facilitate effective workflows. By fostering a culture of mutual learning between AI and healthcare professionals, organizations can empower healthcare professionals while alleviating routine workloads, ultimately improving job satisfaction, well-being, and innovative employee performance. This approach serves as a catalyst for adapting to an increasingly technology-driven and dynamic work environment, ensuring that healthcare organizations remain aligned with contemporary developments in the field.

Second, our research underlines the importance of addressing the psychological needs of healthcare professionals within the medical field. Specifically, the need for autonomy satisfaction, need for competence satisfaction, and need for relatedness satisfaction are identified as critical aspects. It is essential for healthcare organizations to actively support these needs through initiatives such as flexible work arrangements, continuous professional development, and the cultivation of a positive team culture. These measures not only enhance healthcare professionals’ sense of well-being at work but also cultivate a culture of innovation within the profession (Guo et al., 2025). Moreover, hospital administrators should ensure that new healthcare professionals receive adequate resources and organizational support for the integration and application of AI technologies. This support will enable them to adapt their learning capabilities and leverage AI effectively, thereby stimulating their innovative thinking and enhancing their professional abilities.

Third, job complexity serves as a boundary condition that influences the practical utility of intelligent systems in healthcare settings. When addressing high-complexity medical tasks, healthcare organizations must recognize the limitations of AI and encourage healthcare professionals to rely on their professional expertise and judgment in decision-making (Canhoto & Clear, 2020). To better navigate the complexities of the medical environment, institutions should provide necessary support, such as strengthening team cohesion and streamlining work processes. This support will enable healthcare professionals to more effectively manage challenges. Concurrently, continuous advancements in medical AI technology are crucial. By optimizing algorithms and enhancing learning capabilities, AI systems can be better equipped to handle complex cases, thereby offering more robust support to healthcare professionals in diagnostics and treatment.

Moreover, work well-being is a multidimensional concept influenced by a variety of factors (Miao et al., 2024). Healthcare organizations should adopt a holistic approach when developing strategies to enhance the well-being of healthcare professionals. This approach should consider key elements such as optimizing the work environment, fostering positive interpersonal relationships, supporting opportunities for personal and professional growth, and promoting a healthy work-life balance. By implementing these comprehensive measures, organizations can more effectively promote healthcare professionals’ well-being. Such efforts not only benefit individual professionals but also contribute to the overall quality and efficiency of healthcare services, particularly in the context of integrating AI technologies.

### 5.3. Limitations and Directions for Future Research

Our study has certain limitations and offers directions for future research. First, the scope of this study is confined to exploring the impact of the use of AI in healthcare on the work well-being of healthcare professionals. This focus is unique compared to other fields, given the critical nature of life-safety concerns in healthcare, which may limit the applicability of our theoretical model to other professions that also involve emerging technologies (Topol, 2019). As such, the generalizability of our findings is constrained. Future research could broaden this scope by collecting and analyzing data from diverse sectors, such as services, finance, and education, to enhance the generalizability of the results.

Second, although this study employs a time-lagged survey design to mitigate CMB (Piyathasanan et al., 2018), the data are primarily collected through self-reported questionnaires. This methodology does not allow for definitive causal inferences regarding the relationships between the use of AI, psychological needs satisfaction, and work well-being. Future studies could address this limitation by adopting controlled experimental designs or longitudinal data collection to further validate our findings. Additionally, while this research examines the role of job complexity as a moderating factor, it does not fully capture the nuances of the relationship between the use of AI and work well-being. Future research could delve deeper into this relationship by considering variables such as the specialization and personality traits of physicians, as well as the varying levels of AI sophistication (e.g., automation AI vs. augmentation AI) (Guo et al., 2025; Nazareno & Schiff, 2021). This would provide a more comprehensive understanding of how these factors impact the integration of AI in healthcare settings.

Third, our study primarily relies on samples collected from healthcare professionals in Chinese hospitals. As such, the representativeness of the sample may be influenced by regional, cultural context, and national differences in the development of AI, which could cause certain biases. For instance, due to cultural differences, healthcare professionals in collectivist and individualist societies may approach complex medical challenges distinctively. Therefore, future research could benefit from collecting a broader and more diverse sample, including data from various countries. Comparative and cross-cultural analyses would provide a more comprehensive understanding of the specific impacts of medical AI usage on healthcare professionals.

To more comprehensively assess the broader impact of AI in healthcare, future studies could also focus on the patient experience, particularly examining the effects of AI implementation on patient trust and satisfaction. Additionally, the influence of AI on the doctor–patient relationship warrants rigorous investigation, as AI has the potential to significantly alter communication patterns and trust within this critical interaction.

## 6. Conclusions

Drawing on SDT, we develop a conceptual framework to explore the positive impact of the use of AI on healthcare professionals. This framework offers new theoretical insights into the relationship between intelligent machines and healthcare professionals’ work well-being, emphasizing the beneficial effects of AI technologies. Specifically, AI optimizes workflows and improves the efficiency of diagnosing routine or simple conditions. By reducing the time spent on routine tasks, AI allows healthcare professionals to focus on more complex and rewarding tasks, such as diagnosing complex medical conditions.

Moreover, this shift enhances their professional knowledge and capabilities, fostering greater intrinsic motivation and satisfying their psychological needs. As a result, healthcare professionals’ work well-being is further strengthened, enabling them to adapt more effectively to a technology-driven, continuously evolving work environment. Additionally, this study acknowledges the limitations of AI in addressing complex medical tasks. In summary, our research contributes to the literature by addressing the impact of AI on healthcare professionals’ psychological needs and related outcomes, offering valuable insights for the effective integration of AI in healthcare settings.

## Figures and Tables

**Table 1:** Measurement items, reliability, and internal consistency reliability.

Variables and Survey Items	Factor Loading	Cronbach’s α	CR	AVE
**Use of AI (USAI)**		0.878	0.880	0.710
I depend on medical AI to help me with work-related tasks.	0.876			
I collaborate with medical AI to make key work-related decisions.	0.831			
I use AI to review and monitor the quality of my work.	0.819			
**Need for Autonomy Satisfaction (NS_AUT)**		0.755	0.757	0.509
When I collaborate with medical AI, I can still follow my own approach to diagnosis and treatment.	0.724			
When I collaborate with medical AI, I still have a voice and the ability to express my opinions.	0.728			
When I collaborate with medical AI, I feel controlled and pressured to act in a certain way. (R)	0.688			
**Need for Competence Satisfaction (NS_COM)**		0.784	0.784	0.548
When I collaborate with medical AI, I feel capable in my work.	0.757			
When I collaborate with medical AI, I often feel inadequate or incompetent. (R)	0.745			
When I collaborate with medical AI, I feel both capable and efficient.	0.718			
**Need for Relatedness Satisfaction (NS_REL)**		0.803	0.803	0.576
When collaborating with medical AI, I feel as if it cares for and supports me like a colleague.	0.785			
When collaborating with medical AI, I feel I can establish a relationship with it, just like with a colleague.	0.73			
When collaborating with medical AI, I feel a strong sense of closeness and warmth, as if it were a colleague.	0.76			
**Work Well-being (WWB)**		0.888	0.890	0.573
Since the introduction of medical AI, I find my work to be more interesting.	0.757			
Since the introduction of medical AI, overall, I am very satisfied with the work I do.	0.819			
Since the introduction of medical AI, I am always able to find ways to enrich my work.	0.711			
Since the introduction of medical AI, I am generally satisfied with the specific tasks I perform.	0.778			
Since the introduction of medical AI, I feel that my work is a meaningful experience.	0.794			
Since the introduction of medical AI, I am generally satisfied with the sense of accomplishment I gain from my work.	0.672			
**Job complexity (JC)**		0.86	0.841	0.575
My current work tasks are very complex.	0.659			
I have to make very complex decisions in my work.	0.601			
In my work, I need to apply all the knowledge and skills I possess.	0.908			
In my work, I need to continuously learn knowledge related to new things.	0.825			

Note: CR = composite reliability. AVE = average variance extracted. R = Reverse.

**Table 2:** Descriptive statistics and inter-construct correlations.

	Mean	SD	USAI	NS_AUT	NS_COM	NS_REL	WWB	JC	Gender	Age	Edu	Tenure
USAI	3.133	1.130										
NS_AUT	3.652	0.913	0.256 **									
NS_COM	3.639	0.934	0.324 **	0.752 **								
NS_REL	3.457	1.030	0.366 **	0.532 **	0.616 **							
WWB	3.638	0.895	0.284 **	0.597 **	0.557 **	0.460 **						
JC	3.415	1.087	0.406 **	0.306 **	0.302 **	0.313 **	0.314 **					
Gender	1.71	0.456	0.002	0.125 *	0.119 *	0.090	0.060	0.074				
Age	3.070	0.903	0.011	−0.019	−0.050	0.046	0.059	0.047	−0.073			
Edu	2.740	0.903	−0.149 *	0.022	−0.037	−0.113	0.050	−0.008	−0.088	0.153 *		
Tenure	2.850	1.323	0.059	−0.043	−0.069	0.042	0.047	0.113	−0.027	0.710 **	−0.069	

Note: *n* = 280. * *p* < 0.05. ** *p* < 0.01. USAI = Use of AI. NS_AUT = Need for Autonomy Satisfaction. NS_COM = Need for Competence Satisfaction. NS_REL = Need for Relatedness Satisfaction. WWB = Work Well-being. JC = Job Complexity.

**Table 3:** Chi-square, goodness-of-fit values, and model comparison tests.

Model	$\chi^{2}$	df	CFI
1. CFA	318.522	209	0.966
2. Baseline	325.53	215	0.966
3. Method-C	325.009	214	0.966
4. Method-U	290.205	193	0.97
5. Method-R	300.661	209	0.972
Chi-Square Model Comparison Tests			
∆Model	∆$\chi^{2}$	∆df	Chi-Square Critical Value; 0.05
1. Baseline vs. Method-C	0.53	1	3.841
2. Baseline vs. Method-U	35.325 *	22	34.382
3. Method-U vs. Method-R	10.456	16	26.296

Note: * *p* < 0.05. CFA = Confirmatory factor analysis. CFI = Comparative Fit Index. Method-C = Control method variance model. Method-U = Unrestricted method variance model. Method-R proposed by Richardson et al. (2009).

**Table 4:** Discriminant validity and common method bias.

Model	$\chi^{2}$	df	$\chi^{2}/df$	RMSEA	SRMR	TLI	CFI
Six-factor model ^a^	296.107	193	1.534	0.044	0.0488	0.962	0.968
Five-factor model ^b^	308.888	198	1.56	0.045	0.0501	0.96	0.966
Five-factor model ^c^	378.884	198	1.914	0.057	0.0545	0.935	0.944
Four-factor model ^d^	404.5	202	2.002	0.06	0.0571	0.929	0.938
Three-factor model ^e^	692.306	205	3.377	0.092	0.0748	0.831	0.85
Two-factor model ^f^	1009.233	207	4.876	0.118	0.1041	0.725	0.753
One-factor model ^g^	1374.746	208	6.609	0.142	0.1182	0.602	0.641

Note: ^a^ Six-factor model: USAI, NS_AUT, NS_COM, NS_REL, WWB, JC. ^b^ Five-factor model: USAI, NS_AUT + NS_COM, NS_REL, WWB, JC. ^c^ Five-factor model: USAI, NS_AUT, NS_COM + NS_REL, WWB, JC. ^d^ Four-factor model: USAI, NS_AUT + NS_COM + NS_REL, WWB, JC. ^e^ Three-factor model: USAI, NS_AUT + NS_COM + NS_REL + WWB, JC. ^f^ Two-factor model: USAI, NS_AUT + NS_COM + NS_REL + WWB + JC. ^g^ One-factor model: USAI + NS_AUT + NS_COM + NS_REL + WWB + JC. RMSEA = Root Mean Square Error of Approximation. SRMR = Standardized Root Mean Square Residual. TLI = Tucker–Lewis Index. CFI = Comparative Fit Index. USAI = Use of AI. NS_AUT = Need for Autonomy Satisfaction. NS_COM = Need for Competence Satisfaction. NS_REL = Need for Relatedness Satisfaction. WWB = Work Well-being. JC = Job Complexity.

**Table 5:** Results of hypothesis testing.

	**WWB**		**NS_AUT**					
	Model 1	Model 2	Model 3	Model 4	Model 5	Model 6		
Variable	β	β	β	β	β	β		
Gender	0.068	0.070	0.127 *	0.13	0.111	0.111		
Age	0.039	0.041	0.027	0.029	0.044	0.041		
Edu	0.051	0.095	0.0250	0.064	0.045	0.04		
Tenure	0.024	0.009	−0.058	−0.072	−0.106	−0.101		
USAI		0.297 ***		0.269 ***	0.171 **	0.17 **		
JC					0.239 ***	0.212 **		
USAI × JC						−0.13 *		
F	0.697	5.84 ***	1.29	5.352 ***	7.133 ***	6.941 ***		
∆F	0.697	26.158 ***	1.29	21.222 ***	14.698 ***	5.141 *		
R^2^	0.100	0.096	0.018	0.089	0.136	0.152		
∆R^2^	0.100	0.086	0.018	0.071	0.047	0.016		
	**NS_COM**				**NS_REL**			
	Model 7	Model 8	Model 9	Model 10	Model 11	Model 12	Model 13	Model 14
Variable	β	β	β	β	β	β	β	β
Gender	0.115	0.118 *	0.102	0.101	0.085	0.089	0.073	0.073
Age	0.025	0.027	0.04	0.037	0.092	0.094	0.107	0.106
Edu	−0.036	0.012	−0.005	−0.01	−0.122	−0.07	−0.086	−0.087
Tenure	−0.087	−0.104	−0.134	−0.127	−0.029	−0.048	−0.076	−0.075
USAI		0.332 ***	0.247 ***	0.246 ***		0.358 ***	0.276 ***	0.276 ***
JC			0.207 **	0.177 **			0.198 **	0.193 **
USAI × JC				−0.146 *				−0.025
F	1.38	7.98 ***	8.804 ***	8.678 ***	1.729	9.619 ***	10.081 ***	8.644 ***
∆F	1.38	33.723 ***	11.41 **	6.799 *	1.729	40.191 ***	10.69 **	0.202
R^2^	0.02	0.127	0.162	0.183	0.025	0.149	0.181	0.182
∆R^2^	0.02	0.107	0.035	0.02	0.025	0.125	0.032	0.001

Note: *n* = 280. * *p* < 0.05. ** *p* < 0.01. *** *p* < 0.001. USAI = Use of AI. NS_AUT = Need for Autonomy Satisfaction. NS_COM = Need for Competence Satisfaction. NS_REL = Need for Relatedness Satisfaction. WWB = Work Well-being. JC = Job Complexity.

**Table 6:** Results of mediation effects.

				95% Confidence Interval	
Path: USAI -> WWB	Coefficient	T	*p*	LLCI	ULCI
Total effect	0.236	5.114	0.00	0.145	0.326
Direct effect	0.074	1.834	0.068	−0.005	0.154
Indirect effect					
USAI -> NS_AUT -> WWB	0.081			0.033	0.138
USAI -> NS_COM -> WWB	0.046			0.002	0.103
USAI -> NS_REL -> WWB	0.035			0.003	0.073

Note: USAI = Use of AI. NS_AUT = Need for Autonomy Satisfaction. NS_COM = Need for Competence Satisfaction. NS_REL = Need for Relatedness Satisfaction. WWB = Work Well-being. JC = Job Complexity.

**Table 7:** Results for moderated mediator test.

Mediation Variable	Mediator			Index of Moderated Mediation	
	Moderator	Effect	(BootCI)	Index	(BootCI)
NS_AUT	L-JC(−1SD)	0.089	[0.026, 0.170]	−0.035	[−0.077, −0.002]
	H-JC(+1SD)	0.013	[−0.030, 0.059]		
NS_COM	L-JC(−1SD)	0.054	[0.003, 0.129]	−0.018	[−0.051, 0.000]
	H-JC(+1SD)	0.014	[−0.006, 0.047]		
NS_REL	L-JC(−1SD)	0.029	[0.002,0.068]	−0.002	[−0.015, 0.008]
	H-JC(+1SD)	0.024	[0.002, 0.060]		

Note: NS_AUT = Need for Autonomy Satisfaction. NS_COM = Need for Competence Satisfaction. NS_REL = Need for Relatedness Satisfaction. JC = Job Complexity. H = High. L = Low. SD = Standard Deviation. CI = Confidence Interval.

## Data Availability

The data presented in this study are available on request from the corresponding author due to privacy and ethical constraints.

## Note

1 Self-report as a measurement method (it is one of the main sources of measurement error) may introduce common method bias in the collected data, influenced by factors such as respondent consistency, transient emotional states, and other related factors. Collecting data at two different points in time (1 month apart) allows for a more independent, objective, and reliable correlation between the variables (Podsakoff et al., 2003).
